# Seven-Year Follow-up of the RANZCO-Cambodian Ophthalmological Society Partnership CPD Program

**DOI:** 10.5334/aogh.3814

**Published:** 2022-10-11

**Authors:** Heather G. Mack, Lawrence Lee, Helena Prior Filipe

**Affiliations:** 1 Centre for Eye Research Australia, East Melbourne 3002, Australia; 2 Department of Surgery (Ophthalmology), University of Melbourne, 3010, Australia; 3 Department of Ophthalmology, Royal Brisbane and Women’s Hospital and University of Queensland, Brisbane 4006, Australia; 4 Department of Surgery (Ophthalmology), Hospital Egas Moniz, West Lisbon Hospitals Center, Portugal; 5 Department of Medical Education, Faculty of Medicine, University of Lisbon, Portugal

**Keywords:** continuing medical education, continuing professional development, Kirkpatrick, community of practice, LINKS

## Abstract

In 2013 the Royal Australian and New Zealand College of Ophthalmologists partnered with the Cambodian Ophthalmological Society (COS) to develop a continuing professional development program for COS using a college-college twinning model. The program was reviewed seven years after launch. No evidenceof a functioning CPD program was identified. Reasons may include lack of engagement by ophthalmologists and lack of COS resources. A planning checklistfor international CPD collaborations is discussed.

## To the Editor

In 2013, under the auspices of the International Agency for the Prevention of Blindness and Vision2020 Australia East Asia Vision Program, the Royal Australian and New Zealand College of Ophthalmologists (RANZCO) accepted an invitation to partner with the Cambodian Ophthalmological Society (COS) to collaboratively develop a Continuing Professional Education program (CPD) for COS [1], using a college-college twinning model similar to the UK LINKS program [2]. Non-recurring funding was a $114,000 AUD Australian Federal Government AusAID grant, with approximately $100,000 AUD in-kind supplement from RANZCO. A program incorporating competencies required by ophthalmologists and reflective practice was developed [3]. We reviewed the program seven years after the joint RANZCO-COS implementation committee was disbanded as planned at program launch.

CPD programs may be reviewed using a modified Kirkpatrick model where the lowest level measures attendance at CPD events and the highest-level measures improvement in public health outcomes [4], such as cataract surgery rate. Establishment of a community of practice, a group of individuals sharing a common domain of interest and the desire to learn and develop practice, is another useful metric.

We attempted to contact COS via its website (cambodiacos.com) and by email and printed mail to past and present office-bearers. We intended to survey Cambodian eye-care practitioners using the same questionnaire as previously [3] and to perform videoconference focus group interviews. We aimed to review the CPD program outlined on the COS website, and to obtain data from the Cambodian Medical Council on CPD compliance. We intended to obtain statistics on cataract surgery rate in Cambodia.

COS and its office-bearers were not able to be contacted. The COS website was inactive. We have no evidence of development of a community of practice of Cambodian ophthalmologists. The Cambodian Medical Council did not respond to email. We concluded the CPD program to be non-functional, although it is not possible to exclude ophthalmologists undertaking self-initiated and self-recorded CPD activities. Similarly, the LINKS program was not able to develop online CPD, although there was some progress in regional CPD [2].

Likely multiple reasons underpin the apparent failure of the CPD program. Lack of engagement from Cambodian ophthalmologists was identified early as a potential problem and probably continued despite efforts at engagement by educational meetings and instructional workshops [1]. Lack of COS resources was also identified as a potential problem [1]. Undertaking CME activities is mandatory for re-registration of Cambodian medical practitioners, but it is possible that without support and/or enforcement by the CMC, the importance of CPD is not recognised by practitioners. Lack of clarity of educational roles between COS and the University of Health Sciences (Cambodia), and between the multiple non-government organizations in the eye care sector in Cambodia may have also contributed.

Lessons learnt are consistent with the requirements for successful collaboration recommended by Eastwood et al. [5], including the need to fully engage local society leaders with regular contact from both groups, secure long-term funding and the need for long-term collaboration >10 years. Reliable IT systems and efforts to entrench the CPD concept are required, as noted by Mwangi et al. [2]. Enforceable CPD requirement by medical licensure regulators may be required to drive participation. Specific to Cambodia, well-delineated roles between the multiple stakeholders in eye care education are required. The Figure summarises a recommended checklist of requirements for collaborative CPD programs.

**Figure:**
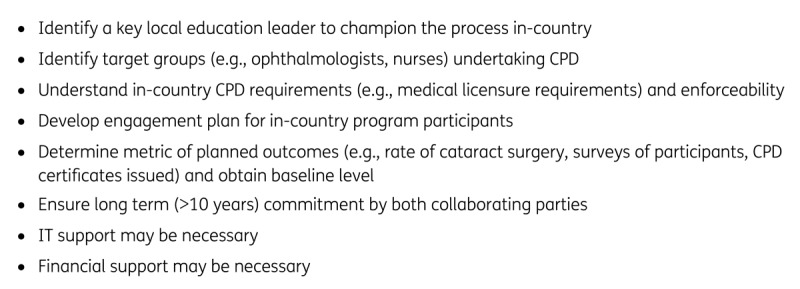
Planning checklist for international CPD collaborations [3,4,5].

More research is necessary to determine optimum methods to establish CPD programs in low resource countries. Development of CPD programs might be better addressed using a ‘greenfield model,’ relying on local leaders to engage members, or, by implementing a regional program designed and implemented collaboratively within bodies such as the Asia-Pacific Academy of Ophthalmology. Ideally all future projects include public health outcomes as a metric.
